# Aspartate β-Hydroxylase (ASPH) Expression in Acute Myeloid Leukemia: A Potential Novel Therapeutic Target

**DOI:** 10.3389/fonc.2021.783744

**Published:** 2021-12-22

**Authors:** Noa G. Holtzman, Michael S. Lebowitz, Rima Koka, Maria R. Baer, Kanam Malhotra, Amir Shahlaee, Hossein A. Ghanbari, Søren M. Bentzen, Ashkan Emadi

**Affiliations:** ^1^ Marlene and Stewart Greenebaum Comprehensive Cancer Center, University of Maryland School of Medicine, Baltimore, MD, United States; ^2^ Immune Deficiency Cellular Therapy Program, Center for Cancer Research, National Cancer Institute, National Institutes of Health, Bethesda, MD, United States; ^3^ Sensei Biotherapeutics Inc., Gaithersburg, MD, United States; ^4^ Department of Pathology, University of Maryland School of Medicine, Baltimore, MD, United States; ^5^ Department of Medicine, University of Maryland School of Medicine, Baltimore, MD, United States; ^6^ Department of Epidemiology and Biostatistics, University of Maryland School of Medicine, Baltimore, MD, United States; ^7^ Department of Pharmacology, University of Maryland School of Medicine, Baltimore, MD, United States

**Keywords:** leukemia, myeloid, myeloblasts, ASPH, leukemia-associated antigen

## Abstract

**Background:**

Aspartate β-hydroxylase (ASPH) is an embryonic transmembrane protein aberrantly upregulated in cancer cells, associated with malignant transformation and, in some reports, with poor clinical prognosis.

**Objective:**

To report the expression patterns of ASPH in acute myeloid leukemia (AML).

**Methods:**

Cell surface expression of ASPH was measured *via* 8-color multiparameter flow cytometry in 41 AML patient samples (31 bone marrow, 10 blood) using fluorescein isothiocyanate (FITC)-conjugated anti-ASPH antibody, SNS-622. A mean fluorescent intensity (MFI) of 10 was used as a cutoff for ASPH surface expression positivity. Data regarding patient and disease characteristics were collected.

**Results:**

ASPH surface expression was found on AML blasts in 16 samples (39%). Higher ASPH expression was seen in myeloblasts of African American patients (p=0.02), but no correlation was found between ASPH expression and other patient or disease characteristics. No association was found between ASPH status and CR rate (p=0.53), EFS (p=0.87), or OS (p=0.17).

**Conclusions:**

ASPH is expressed on blasts in approximately 40% of AML cases, and may serve as a new therapeutically targetable leukemia-associated antigen.

## Introduction

Aspartate β-hydroxylase (ASPH) is an α-ketoglutarate-dependent dioxygenase that promotes cellular growth, motility and adhesion by post-translational hydroxylation of aspartyl and asparaginyl residues in epidermal growth factor-like protein domains, including Notch, Notch homologs, Jagged and extracellular matrix molecules such as laminin and tenascin (1, 2). ASPH is encoded by the aspartate beta-hydroxylase (*ASPH*) gene on chromosome 8 and is upregulated by insulin and insulin growth factor 1 (IGF1) through the MAPK/ERK and PI3K/AKT pathways (2, 3). ASPH can lead to carcinogenesis by inducing decreased cleavage of caspase-3, causing inhibition of apoptosis (4), and may also promote tumor immune escape *via* inhibition of natural killer (NK)-cell activity (5).

ASPH is highly expressed during fetal development and is aberrantly upregulated in cancer cells (6). ASPH is overexpressed in over 20 different solid neoplasms, including liver (7–10), breast (11), lung (12), brain (13), pancreatic (14), gastric (15) and colorectal cancers (16), in which it propagates a malignant phenotype, associated with increased cell proliferation, invasiveness, metastasis, and also with poor clinical prognosis (7, 8, 17–19).

ASPH has been shown to be immunogenic in preclinical studies exploring its role as a target for vaccination (20, 21) and for dendritic cell therapy (22), making it a promising immunotherapeutic target (23–25). ASPH is currently being targeted with an anti-ASPH nanoparticle vaccine, SNS-301, in a clinical trial in prostate cancer (26).

Despite the established role of ASPH in solid neoplasms, little is known about its role in hematologic malignancies. Acute myeloid leukemia (AML) is a heterogeneous hematologic malignancy with an incidence of 4.3 per 100,000 in the US and a 5-year survival rate of only 24% (27). New treatment options are needed for AML and much interest has shifted toward immunotherapeutic strategies in treatment of AML, necessitating identification of leukemia-associated antigens. Studies have demonstrated overexpression of ASPH in the AML cell line MOLM-14 *in vitro*, and successful targeting of myeloblasts with anti-ASPH radiolabeled or cytotoxin-linked antibody drug-conjugates (ADCs) (28, 29).

ASPH may have the potential to serve as a tumor- or leukemia-associated antigen for immunotherapy. In an effort to further characterize ASPH as a therapeutic target in AML, we report here the first study of ASPH expression in AML patient samples.

## Materials and Methods

### Patient Samples

Bone marrow (BM) aspirate and peripheral blood (PB) samples were collected from AML patients treated at the University of Maryland Greenebaum Comprehensive Cancer Center (2014-2018) on a University of Maryland School of Medicine (UMSOM) Institutional Review Board (IRB)-approved institutional tissue procurement protocol. Mononuclear cells isolated by density centrifugation were viably cryopreserved at -80°C in RPMI 1640 with 20% fetal calf serum and 5% dimethyl sulfoxide (DMSO). The study was approved by the UMSOM IRB and was conducted in accordance with the principles of the Declaration of Helsinki.

### ASPH Expression

AML BM and PB samples were analyzed for cell surface expression of ASPH with the fluorescein isothiocyanate (FITC)-conjugated anti-ASPH antibody SNS-622 using 8-color flow cytometry on a MACSQuant^®^ Analyzer 10 flow cytometer (Miltenyi Biotec GmbH, Germany). Two panels of 8 antibodies were used. Panel 1: FITC-SNS-622, VioBlue-CD45, VioGreen-CD33, PE-CD34, PEVio770-CD19, APC-CD13 or APC-CD38, APCVio770-CD117. Panel 2: FITC-SNS-622, VioBlue-CD45, VioGreen-CD20, PE-CD34, PEVio770-HLA-DR, APC-CD3, APCVio770-CD64 (Miltenyi Biotec GmbH, Germany). PE-CD14 and APCVio770-CD64 were substituted in Panel 2 for AMLs with monocytic lineage. Propidium iodide (PI) PerCP770 (Miltenyi Biotec GmbH, Germany) was used in both panels to assess cell viability. The original immunophenotype of the blast population as determined by hematopathologist review in the University of Maryland Pathology Laboratory [certified by Clinical Laboratory Improvement Amendments (CLIA) and accredited by the College of American Pathologists (CAP)] using CD45, CD34, CD13, CD33, CD117, HLA-DR, CD14, CD64 antibodies to define the blast population in each case. Expression of ASPH was analyzed by two independent reviewers using *FlowLogic™* software (Inivai Technologies, Melbourne, Australia).

### Clinical Data

Data regarding patient and disease characteristics, including karyotype and myeloid mutations, treatment and outcomes were collected (**Table 1**). Risk category was classified as favorable, intermediate, or unfavorable based on European LeukemiaNet (ELN) 2017 criteria (30).

**Table 1:** Patient and disease haracteristics.

Patient & Disease Characteristics	Total (n = 41)	Positive ASPH (n = 16)	Negative ASPH (n = 25)	*p-value*
**Age, y median (range)**	66 (18-87)	62.5 (18-87)	67.0 (30-82)	p = 0.24
**Sex, n (%)**				
Female	20 (49)	8 (50)	12 (48)	p = 1.0
Male	21 (51)	8 (50)	13 (52)	
**Ethnicity, n (%)**				
Caucasian	28 (68)	7 (44)	21 (84)	**p = 0.02**
African American	9 (22)	7 (44)	2 (8)	
Asian	3 (7)	2 (12.5)	1 (4)	
Other	1 (2)	0 (0)	1 (4)	
**Disease State, n (%)**				
Untreated *de novo*	20 (49)	9 (56)	11 (44)	p = 0.28
Untreated secondary (antecedent MDS or MPN)	8 (20)	3 (19)	5 (20)	
**Relapsed or Refractory**	13 (31)	4 (25)	9 (36)	
**Risk Category (ELN), n (%)**				
Favorable	2 (5)	0 (0)	2 (8)	p = 0.25
Intermediate	8 (20)	5 (31)	3 (12)	
Unfavorable	31 (76)	11 (69)	20 (80)	
**Karyotype, n (%)**				
Normal	21 (51)	9 (56)	12 (48)	p = 1.00
Complex	6 (15)	2 (13)	4 (16)	
Other	14 (34)	5 (31)	9 (36)	
**FAB subtype, n (%)**				
Monocytic (M4, M5)	19 (46)	9 (56)	10 (40)	p = 0.19
Non-monocytic (M0,M1,M2,M6,M7)	20 (49)	5 (31)	15 (60)	
N/A	2 (5)	2 (13)	0 (0)	
**Mutations, n (%)**				
***FLT3*-ITD or *FLT3*-TKD**	17 (41)	7 (44)	10 (40)	
***NPM1***	12 (29)	6 (38)	6 (24)	
***ASXL1***	6 (15)	3 (19)	3 (12)	
***TP53***	4 (10)	1 (6)	3 (12)	
**Induction regimen, n (%)**				
7+3 backbone	27 (66)	12 (75)	15 (60)	p = 0.50
Hypomethylating agent	14 (34)	4 (25)	10 (40)	

the p-value that is in bold is statistically significant.

Treatment outcomes included (1) complete remission (CR) - defined as less than 5% bone marrow blasts (as detected by immunohistochemistry on core biopsy) and no evidence of disease elsewhere with packed red blood cell transfusion independence, absolute neutrophil count (ANC) ≥1x10^9^/L and platelet count ≥100x10^9^/L; (2) CRi – fulfills criteria for CR but with incomplete count recovery, or (3) induction failure. Overall survival (OS) was calculated from the time of diagnosis to the time of death or censored at the time of last contact in patients still alive. Event-free survival (EFS) was calculated from the time of AML diagnosis until induction failure, relapse, or death from any cause; patients alive without disease were censored at the time of last follow-up.

### Statistical Analysis

Statistical analysis was conducted using IBM^®^ SPSS^®^ Statistics for Windows, release 25.0.0 (IBM Corp., Armonk, N.Y., USA). Visual inspection of the distribution of ASPH expression data on myeloblast or monoblast surface within the whole cell population was used to identify a robust cut-point separating high (i.e., positive) from low (i.e., negative) ASPH expression. Cohen’s kappa (k) was used to quantify the agreement between two independent observers on classification into ASPH-positive vs. -negative disease. The distribution of ASPH positivity in various subgroups of patients was analyzed using Pearson’s chi-square test or, for 2x2 tables, Fisher’s exact test. OS and EFS were estimated using the Kaplan-Meier estimator and compared between ASPH-positive and -negative groups using the Mantel-Cox log-rank test.

## Results

### Patient Characteristics

AML patients were evaluated, including 31 BM and 10 PB (**Table 1**). Median patient age was 66 years (range, 18-87 years), 49% (n=20) were female, 68% (n=28) Caucasian and 22% (n=9) African American. Disease status was untreated *de novo* in 49%, untreated secondary from an antecedent myelodysplastic syndrome (MDS) or myeloproliferative neoplasm (MPN) in 20%, and relapsed/refractory in 31%. Samples were cytogenetically and molecularly diverse, including 51% with normal karyotype and 15% with complex karyotype; 41% had FMS-like tyrosine kinase 3 internal tandem duplication (*FLT3*-ITD) or *FLT3* tyrosine kinase domain (TKD) mutations, 29% nucleophosmin 1 (*NPM1*), and 10% *TP53* mutations. Most patients (76%) had unfavorable risk disease per ELN-2017 risk criteria. Induction therapy for 66% of patients included a 7 + 3 cytarabine plus anthracycline backbone, and the remainder (34%) were treated with hypomethylating agents (HMAs; azacitidine or decitabine). Fourteen patients (34%) underwent consolidative allogeneic hematopoietic stem cell transplant (HSCT) (**Table 2**).

**Table 2:** Treatment response and clinical outcomes.

	Total (n = 41)	ASPH-positive (n = 16)	ASPH-negative (n = 25)
**CR/CRi, n (%)**	20 (49)	9 (56)	11 (44)
**Primary Refractory Disease, n (%)**	16 (39)	6 (38)	10 (40)
**Disease Relapse, n (%)**	18 (44)	7 (44)	11 (44)
**HSCT, n (%)**	14 (34)	8 (50)	6 (24)
**Relapse post-HSCT, n (%)**	5 (12)	2 (13)	3 (12)

### ASPH Expression

**Figure 1:**
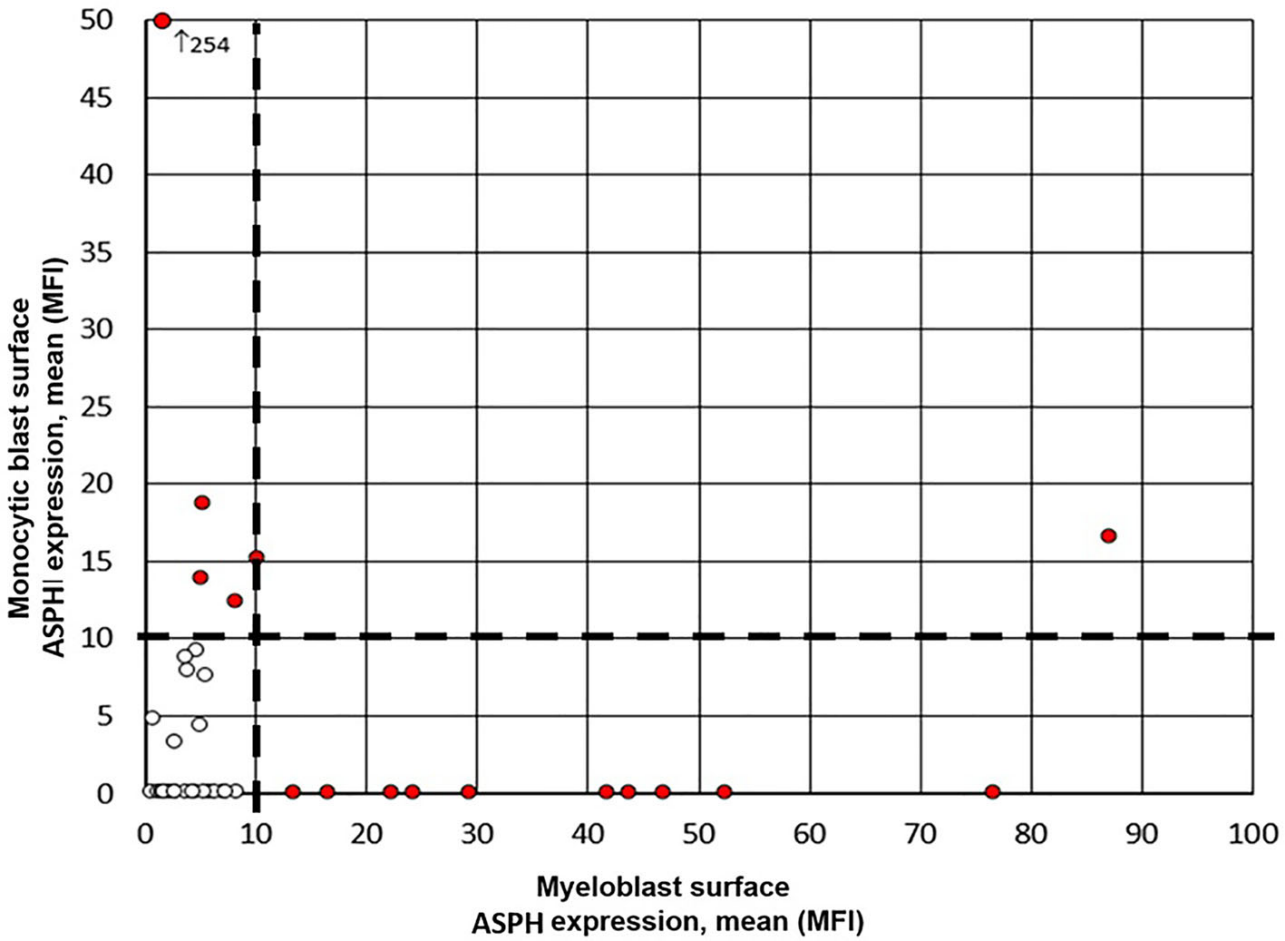
ASPH surface expression on myeloblasts and monoblasts. Expression data for the whole patient population showed a ‘mixed population’, i.e. a clustering of patients with low or zero expression mixed with patients with stronger expression, which was used to identify a robust cut-point of 10 MFI (black dashed line) separating high (i.e., positive, red circles) from low (i.e., negative, white circles) mean ASPH expression on myeloblast (x-axis) or monoblast (y-axis) surface.

ASPH was expressed on AML blasts in 39% of samples (n=16; 13 BM, 3 PB). Expression data for the whole patient population showed a ‘mixed population’, i.e. a clustering of patients with low or zero expression mixed with patients with stronger expression, which determined a mean fluorescent intensity (MFI) of 10 as the cutoff for ASPH surface expression positivity, identifying a robust cut-point separating high (i.e., positive) from low (i.e., negative) ASPH expression (**Figure 1**). Blinded independent review of the data produced a Cohen’s κ of 0.74 (SE of +/- 0.11).

ASPH expression was seen only on the surface of blasts, and not on any other cells. Thirteen patients had two populations of blasts as a characteristic of their disease, both myelo- and monoblasts. ASPH expression was higher in monoblasts than in myeloblasts in 11 of these paired values. This difference was statistically significant (p=0.03, Wilcoxon Signed Rank Test). The mean expression per MFI was 27.3 in monoblasts vs 14.1 in myeloblasts in these 13 cases. Patients with AML with ASPH expression were clinically heterogeneous. Higher ASPH expression was seen in myeloblasts of African American patients in our cohort (p=0.02). No correlation was found between ASPH expression and the following variables: sex, ELN risk category, cytogenetics, *de novo* versus secondary AML classification, monocytic FAB status, and type of induction therapy (**Table 1**).

### Clinical Outcomes

**Figure 2:**
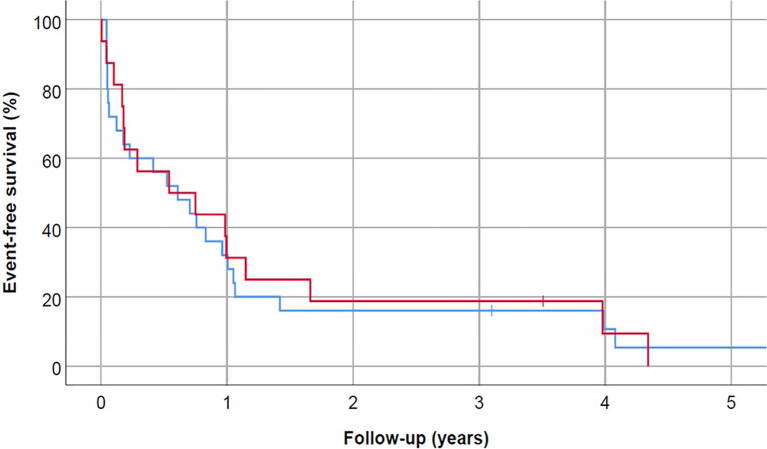
Event-free survival did not correlate with ASPH status (p=0.87). Blue line represents ASPH- negative AML patients and red line is ASPH-positive AML patients.

Twenty patients (49%) achieved CR or CRi, including 9 (56%) with ASPH expression. No association was found between ASPH status and achievement of CR (p = 0.53). Sixteen patients (39%) had primary refractory disease, including 6 (38%) with ASPH expression (**Table 2**). Median EFS was 7.3 months, with no difference based on ASPH status (median EFS for ASPH-positive vs. ASPH-negative patients, 6.5 vs. 7.3 months, p=0.87) (**Figure 2**). When stratified by disease state at diagnosis, among the 13 patients with relapsed/refractory (R/R) disease, 31% (n=4) were ASPH positive (**Table 1**). ASPH positivity was not significantly associated with OS (p=0.71) or EFS (p=0.85) among the R/R cohort. Among 28 patients with newly diagnosed or secondary AML, 43% (n=12) were ASPH positive. Again, in this subgroup analysis, ASPH status was also not significantly associated with OS (p=0.16) or EFS (p=0.39).

**Figure 3:**
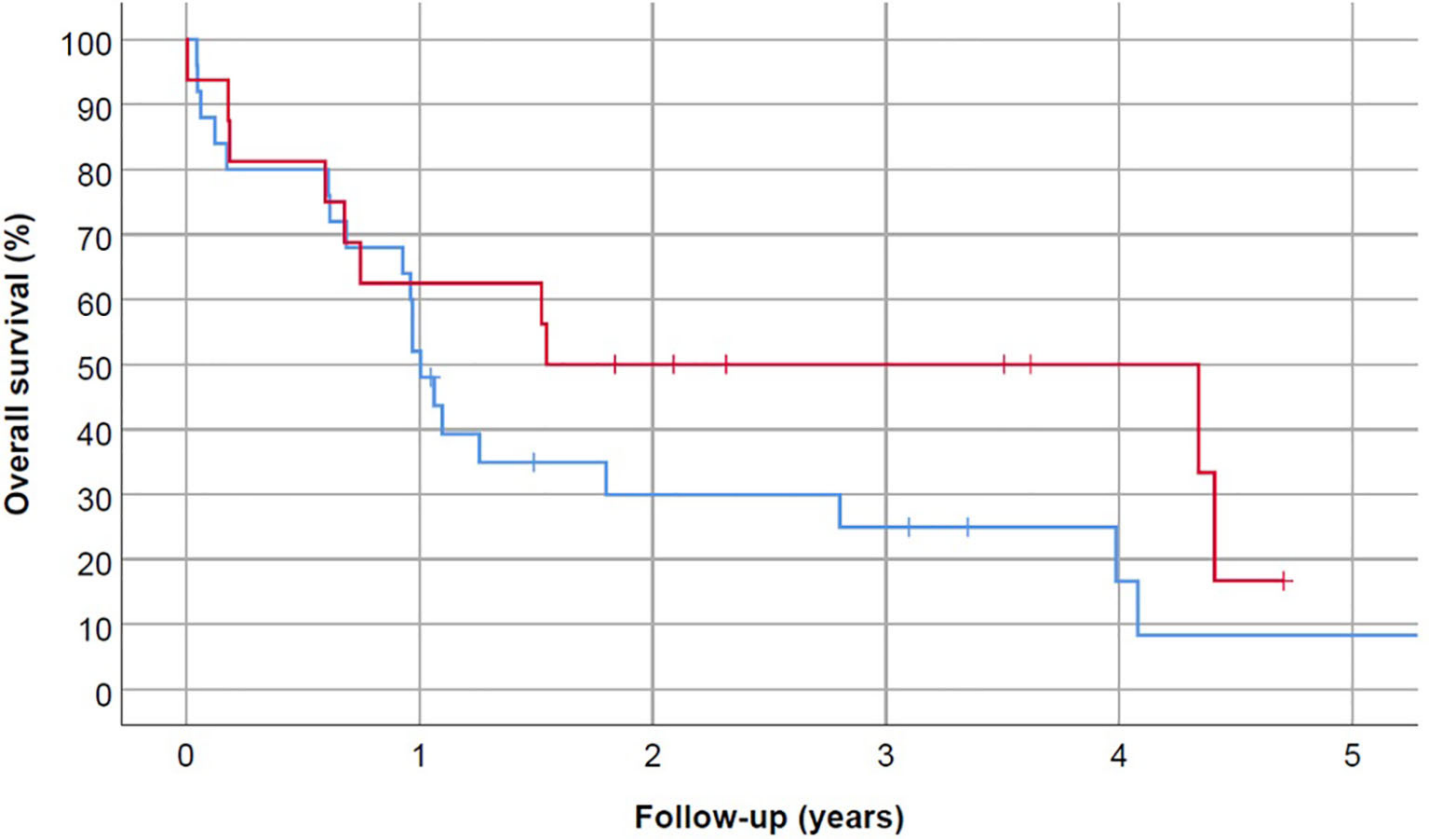
Overall survival did not correlate with ASPH status (p=0.169). Blue line represents ASPH-negative AML patients and red line is ASPH-positive AML patients. Patients were censored at the time of death, loss to follow-up, or HSCT.

Median OS for our cohort was 13.1 months, with no difference based on ASPH expression (median OS for ASPH-positive vs. ASPH-negative, 18.5 vs. 12.0 months, p=0.169) (**Figure 3**). At the time of analysis, 9 patients (22%) were alive, including 6 (67%) who underwent HSCT.

## Discussion

ASPH was overexpressed on blasts of approximately 40% of AML patients in this series. While not appearing to be associated with any specific disease features or with clinical prognosis, this surface expression serves as a potential new therapeutic target for AML.

Discovery of new therapeutic targets in AML remains imperative. Specifically, new treatments are needed for patients with relapsed/refractory disease or those who are unfit for intensive cytotoxic chemotherapy. Immunotherapeutic strategies using ADCs, bispecific T-cell engagers (BiTEs), and chimeric antigen receptor (CAR) T-cell therapy have shown great success in B-cell lymphoid leukemia and lymphoma due to the consistently expressed, safe and effective targets CD19 and/or CD22 (31–34). However, progress for AML has lagged because of lack of a selective leukemia-associated target. Therefore, much interest has been focused on discovery of new tumor- or leukemia-associated antigens, such as ASPH.

ASPH is a safe therapeutic target due to its inherent embryonic function, which is not required for healthy adult human function (barring its possible role in pregnancy) (35, 36). Mutations in the *ASPH* gene lead to Traboulsi Syndrome in humans, reported in four families in the literature. This syndrome is characterized by developmental defects including facial dysmorphism and ophthalmologic abnormalities such as lens dislocation and anterior-segment abnormalities. Most importantly, monitoring of these patients has shown that the loss of ASPH function has not been associated with disease later in life (37).

ASPH is further a promising target due to its immunogenicity. When expressed, ASPH is found at high levels on the cell surface plasma membrane, where its N-terminal sequences and the catalytic site located in the C-terminal region have been shown to serve as antigens that are accessible to the immune system (21). In preclinical studies, vaccination is capable of inducing ASPH epitope-specific CD4^+^ and CD8^+^ T-cell responses in both animal and human models of hepatocellular carcinoma (26). Dendritic cell vaccination studies in a rodent model of biliary cancer showed successful passive vaccination with naïve dendritic cells that were matured *in vivo* in the presence of ASPH, leading to tumor regression, along with ASPH-specific T-cell responses (25). ASPH is also thought to lead to immune surveillance escape and promotes tumor growth by direct effects on NK-cells, including reducing NK-cell viability and cytotoxicity (5).

Importantly, unlike data in solid tumors that suggest the role of ASPH as a predictive marker of poor clinical prognosis (17–19), we found that ASPH status was not associated with clinical outcomes (CR, EFS, OS) in AML. Further, there was no association seen between ASPH status and cytogenetic abnormality, mutation status, or ELN risk category, though notably the majority (74%) of our patient cohort fell into the unfavorable risk category. This may reflect the limitations of our study, including the relatively small patient cohort and the retrospective nature of our clinical outcome review, along with the significant heterogeneity of AML as a clinical entity. The heterogeneity of AML as seen by various combinations of cytogenetic and molecular characteristics that define disease subsets and control how aggressive the disease can be, is important to consider, as due to the small cohort in this study, this heterogeneity may explain the lack of association found with any significant outcome and begs for further large scale studies. Also, changes can be perhaps seen longitudinally in serial samples of the same patient after treatment, where AML myeloblasts can exhibit changes to surface antigen expression and clonal evolution at times, which would be another interesting angle to explore in future trials and not represented by our study that only included samples at time of diagnosis. Additionally, a further limitation may be that while CR data was available for all patients, measurable residual disease (MRD) status at time of CR based on molecular next-generation sequencing was not available for all given the time period of sample collection, which was partly in the years prior to increasing standardization of MRD and its currently established influence on clinical outcomes.

One hypothesis is that while ASPH plays an important role in cancer cell metastasis in solid neoplasms, including cellular detachment, migration, and adhesion at a distant site (19), circulating tumor cells such as leukemic blasts do not need a seeding advantage in order to exert their deleterious effects that lead to poor prognosis. Further investigation regarding a possible relationship between ASPH expression and extramedullary disease in AML would be interesting to explore in future studies. While higher expression of ASPH has been reported in some cancer stem cells such as glioma stem cells (38), its expression on leukemia stem cells (LSC) is not well described. Another question of interest in study of ASPH in leukemia, specifically, includes whether ASPH is differentially expressed in leukemia stem cells compared to more differentiated leukemia blasts, and while this study did not address this question – there has been much study into genes that play a role in stemness for AML. One such study pursuing the development of predictive and/or prognostic biomarkers related to stemness, Ng et al. generated a list of genes that are differentially expressed between 138 LSC positive (LSC+) and 89 LSC− cell fractions from 78 AML patients validated by xenotransplantation. They generated a 17-gene LSC score (LSC17). ASPH was not among the 17 signature genes, which were GPR56, AKR1C3, CD34, NGFRAP1, EMP1, SMIM24, SOCS2, CPXM1, CDK6, KIAA0125, DPYSL3, MMRN1, LAPTM4B, ARHGAP22, NYNRIN, ZBTB46, and DNMT3B. Of note, the LSC17 score was highly prognostic in five independent cohorts of approximately 900 AML patients (39).

An additional potentially important finding was the statistically significant higher incidence of ASPH expression on blasts of African American AML patients. It is unknown if this is a pattern seen across other cancers with ASPH expression. Though our relatively small cohort is a limiting factor, this finding should be further explored prospectively and validated. Racial disparities and their effect on clinical outcomes for AML represent an important field of interest that requires more investigation (40, 41).

Lastly, it has been reported that embryonic antigens, such as ASPH, are upregulated by HMAs (42–44). In our patient cohort, approximately one third (34%) of patients who were treated with HMA (alone or in combination with another drug) had ASPH-positive AML at diagnosis. Among these 14 patients, 4 (29%) were secondary AML patients, 4 (21%) were relapsed/refractory AML, and 7 (50%) were newly diagnosed AML. Importantly, for all these patients, the HMA was administered after the collection of the sample that was analyzed for ASPH expression – none received HMA prior. While the decision to use HMAs likely reflects patients’ older age, poor performance status or AML features, such as complex karyotype, HMA use and potential effects on ASPH expression warrant further investigation. Future studies exploring ASPH expression on serial samples in patients who were treated with or without HMAs would be of great interest. Further, there is potential for synergy when using HMAs as they may increase ASPH antigen expression to allow for concurrent targeting of surface ASPH expression with an immunotherapeutic agent.

In summary, ASPH is overexpressed in approximately 40% of AML cases, and can serve as a potential immunotherapeutically targetable tumor-specific antigen. An anti-ASPH nanoparticle vaccine is currently under clinical investigation, having completed Phase 1 testing with encouraging results in solid tumors (29). Additional immunotherapies such as ADCs and CAR T-cells targeting ASPH may be promising potential therapeutic agents for AML.

## Data Availability Statement

The raw data supporting the conclusions of this article will be made available by the authors, without undue reservation.

## Ethics Statement

The studies involving human participants were reviewed and approved by University of Maryland School of Medicine (UMSOM) Institutional Review Board (IRB). The patients/participants provided their written informed consent to participate in this study.

## Author Contributions

Conception: NH, AE, ML, and HG. Interpretation or analysis of data: NH, ML, AE, RK, MB, and SB. Manuscript preparation: NH, AE, ML, RK, MB, KM, AS, HG, and SB. Revision for important intellectual content: NH, AE, ML, RK, MB, and HG. Supervision: AE, ML, RK, and KM. All authors contributed to the article and approved the submitted version.

## Funding

This research was supported by funds through the National Cancer Institute–Cancer Center Support Grant (CCSG)–P30CA134274.

## Conflict of Interest

Authors ML, KM and AS were employed by company Sensei Biotherapeutics.

ML: Stock ownership in Sensei. AS: Stock ownership in and received consultancy fee from Sensei. HG: Founder and emeritus CSO, Panacea Pharmaceuticals/Sensei Biotherapeutics, Founder/CEO, Athanor Biosciences, MD.

The remaining authors declare that the research was conducted in the absence of any commercial or financial relationships that could be construed as a potential conflict of interest.

## Publisher’s Note

All claims expressed in this article are solely those of the authors and do not necessarily represent those of their affiliated organizations, or those of the publisher, the editors and the reviewers. Any product that may be evaluated in this article, or claim that may be made by its manufacturer, is not guaranteed or endorsed by the publisher.
